# Technical evaluation of an allergen Challenge Theatre^TM^

**DOI:** 10.1186/1710-1492-10-S2-A22

**Published:** 2014-12-18

**Authors:** Suzanne Kelly, Jimmy Yang, Rob Perrins, Jacob Karsh, William H Yang

**Affiliations:** 1 Red Maple Trials Inc., Ottawa, Ontario, Canada

## Background

Allergen challenge chambers expose allergen-sensitive subjects to a predetermined concentration of allergen in a closed, controlled environment and provide a mechanism to induce clinical symptoms and measure the effect of medication.

## Methods

A technical evaluation of the capabilities of the Red Maple Trials Allergen Challenge Theatre^TM^ was performed. The theatre is a 4-zone facility holding up to 99 seats in a series of elevated rows. Grass (*Phleum pratense*) and ragweed (*Ambrosia artemisiifolia*) pollens were injected into the air supply and blown into the facility through ducts located across the top of the front wall. Grass and ragweed pollen concentrations were measured on impact samplers set at face level in 5 sections of a T-shaped quadrant. Concentrations were measured every 30 minutes for 150 minutes. Continuous pollen counts were also read by a laser particle counter (LPC) set to read particles > 5µm and positioned 5 feet above floor level.

## Results

The impact sampler pollen concentration for the theatre quadrant during the entire 180-minute exposure was 3992 ± 975 grains m^3^. Concentrations for the quadrant were consistent at each 30-minute measurement with means ranging from 3648 to 4523 and SDs from 678 to 1105. Pollen concentrations were consistent in each of the 5 sections of the quadrant over time with means ranging from 3112 to 5268 and SDs ranging from 308 to 926. Pollen counts measured by LPC remained consistent at 4000 per m^3^ during the experiment. There was a linear relationship between the LPC pollen readings and the impact sampler readings.

## Conclusions

The Red Maple Trials allergen exposure theatre demonstrated the capacity to achieve and maintain a concentration of pollen grains at a magnitude consistent with the literature and associated with the ability to induce symptoms of appropriate intensity upon allergen challenge. The use of an LPC provided a significant advantage by monitoring pollen counts on a continuous basis. The chamber with a seating capacity of 99 places has the ability to evaluate large test groups at a time.

